# Avicularin and Senegenin Suppress TRPV3-Associated TSLP Expression in Human Keratinocytes

**DOI:** 10.3390/cells15181659

**Published:** 2026-09-14

**Authors:** Han Bi Kim, Ji Young Um, Da Eun Song, Bo Young Chung, Chun Wook Park, Hye One Kim

**Affiliations:** Department of Dermatology, College of Medicine, Hallym University, Kangnam Sacred Heart Hospital, 1 Singil-ro, Seoul 07441, Republic of Korea; khmamy1029@naver.com (H.B.K.); ujy0402@hanmail.net (J.Y.U.); sde1026@naver.com (D.E.S.); victoryby@naver.com (B.Y.C.); dermap@hanmail.net (C.W.P.)

**Keywords:** Avicularin, calcium signaling, keratinocytes, Senegenin, thymic stromal lymphopoietin (TSLP), transient receptor potential vanilloid channel 3 (TRPV3)

## Abstract

**Highlights:**

**What are the main findings?**

- Avicularin (AVC) and senegenin (SNG) attenuate carvacrol-induced calcium-dependent fluorescence responses in TRPV3-expressing cells.
- AVC and SNG attenuate NFAT/NF-κB signaling without additive effects in combination with pathway inhibitors, supporting convergence of their effects with TRPV3-associated NFAT/NF-κB–TSLP signaling.

**What are the implications of the main findings?**

- These findings identify AVC and SNG as promising natural modulators of TRPV3-associated inflammatory signaling and provide mechanistic support for their anti-inflammatory activity in keratinocytes.
- Targeting the TRPV3–TSLP signaling pathway with plant-derived compounds may represent a promising approach for further investigation in pruritic inflammatory skin disorders, including atopic dermatitis.

**Abstract:**

Transient receptor potential vanilloid channel 3 (TRPV3) is a keratinocyte-expressed ion channel that regulates calcium signaling and thymic stromal lymphopoietin (TSLP) production, contributing to pruritic inflammatory skin diseases. Although avicularin (AVC), a flavonoid from *Polygonum aviculare* L., and senegenin (SNG), the bioactive aglycone of *Polygala tenuifolia* Willd., possess anti-inflammatory properties, their effects on TRPV3-associated signaling remain unknown. A natural product library was screened for nitric oxide (NO) production in carvacrol-stimulated human keratinocytes. Following primary screening and secondary evaluation of TRPV3 and TSLP expression, AVC and SNG were selected for further study and subsequently evaluated by molecular docking, calcium imaging, quantitative real-time PCR, immunocytochemistry, and pathway inhibition assays. AVC and SNG showed favorable predicted docking poses at both TRPV3 docking sites and significantly attenuated carvacrol-induced calcium-dependent fluorescence responses in TRPV3-expressing cells. Both compounds reduced TRPV3 and TSLP expression at the mRNA and protein levels and attenuated the nuclear translocation of phospho-Nuclear Factor of Activated T Cells 2 (NFATC2) and phospho-p50. Co-treatment with NFAT or Nuclear Factor Kappa B (NF-κB) inhibitors produced no additional suppression, consistent with convergence of AVC/SNG-associated effects with NFAT/NF-κB signaling. Collectively, these findings support further investigation of AVC and SNG as candidate modulators of TRPV3-associated inflammatory signaling in keratinocytes.

## 1. Introduction

Pruritic inflammatory skin diseases, including atopic dermatitis (AD), chronic urticaria, prurigo, and psoriasis, represent a major global health burden, collectively ranking among the leading causes of years lived with disability worldwide [1]. These conditions are characterized by persistent itch, skin barrier dysfunction, and chronic immune activation, and they are often associated with elevated levels of pro-inflammatory cytokines and neuropeptides [2]. Despite advances in understanding the pathophysiology of these disorders, effective and well-tolerated long-term therapies remain limited, underscoring the need to identify novel molecular targets and candidate compounds.

Transient receptor potential vanilloid 3 (TRPV3) is a thermosensitive cation channel belonging to the TRP superfamily that is highly expressed in epidermal keratinocytes [3]. Each subunit comprises six transmembrane domains (S1–S6) with an intracellular N- and C-terminus and a pore-forming loop between S5 and S6, and four subunits assemble into a functional homotetramer [4]. The S1–S4 (voltage-sensor-like domain [VSLD]) constitutes a distinct allosteric regulatory site that mediates ligand-dependent channel modulation independently of the pore domain [4]. TRPV3 is activated by warm temperatures (33–39 °C), as well as by chemical ligands including carvacrol, thymol, and eugenol [5]. Upon activation, TRPV3 mediates calcium (Ca^2+^) influx into keratinocytes, triggering downstream signaling cascades that promote the release of inflammatory mediators [6]. TRPV3-mediated Ca^2+^ entry drives nitric oxide (NO) production in keratinocytes via nitrite-dependent pathways [7] and activates NF-κB signaling, leading to upregulation of inducible nitric oxide synthase (iNOS) [8]. These downstream consequences of TRPV3 activation provided the mechanistic basis for employing NO inhibition as the primary functional readout in our natural product library screen. Given the large number of compounds screened, NO production was used as a practical primary screening readout, with cell viability assessed in parallel to identify potential cytotoxic effects. TRPV3 and thymic stromal lymphopoietin (TSLP) expression were subsequently evaluated to prioritize candidates for further mechanistic investigation.

Beyond thermosensation, TRPV3 plays roles in skin barrier formation, keratinocyte differentiation, and hair follicle cycling [9,10]. Pathologically, gain-of-function mutations in TRPV3 cause Olmsted syndrome, a rare genodermatosis characterized by severe palmoplantar keratoderma and pruritus [11], and TRPV3 expression is elevated in lesional skin of AD patients, correlating with itch severity and barrier dysfunction [12]. Notably, TRPV3 activation has been shown to induce thymic stromal lymphopoietin (TSLP) production in epidermal keratinocytes [13,14]. TSLP is a pleiotropic epithelial-derived cytokine that plays a critical role in initiating and perpetuating type 2 inflammatory responses associated with itch and atopic skin disease [15,16].

TSLP acts on dendritic cells, mast cells, innate lymphoid cells, and sensory neurons to promote Th2 skewing and sensitization of itch-sensing nerve fibers [17]. The TRPV3–TSLP signaling axis has therefore emerged as a compelling therapeutic target for pruritic inflammatory conditions. Several small-molecule TRPV3 antagonists have been developed and evaluated in preclinical models [18]; however, concerns regarding systemic toxicity, poor bioavailability, and limited selectivity have hindered their clinical translation. Natural products represent an attractive alternative given their structural diversity, favorable safety profiles, and established biological activity [19].

AVC (quercetin-3-O-α-L-arabinofuranoside) is a flavonoid glycoside naturally present in *Polygonum aviculare* L., a plant used in traditional Korean herbal medicine for the treatment of inflammatory diseases, including arthritis and skin disorders [20,21]. In functional studies, AVC has been shown to suppress lipopolysaccharide (LPS)-induced production of pro-inflammatory mediators, including NO and prostaglandin E2 (PGE2), and to inhibit iNOS and cyclooxygenase-2 (COX-2) protein expression in macrophages [22]. AVC has also been shown to reduce the expression of inflammatory cytokines, including interleukin (IL)-1β, IL-6, IL-8, and matrix metalloproteinases (MMP-1 and MMP-13) in tumor necrosis factor-α (TNF-α)-stimulated cells [23].

SNG [(2β,3β,4α,12α)-12-(chloromethyl)-2,3-dihydroxy-27-norolean-13-ene-23,28-dioic acid; also known as tenuigenin] is a pentacyclic triterpenoid sapogenin and the key bioactive aglycone metabolite of *Polygalae Radix*—the dried roots of *Polygala tenuifolia* Willd. (Yuan Zhi), a plant prescribed in Traditional Chinese Medicine (TCM) for centuries to enhance memory and treat neurological and psychiatric conditions including insomnia, depression, and dementia [24]. Notably, extracts of P. tenuifolia have been demonstrated to alleviate stress-exacerbated atopy-like skin dermatitis in murine models by modulating protein kinase A (PKA) and p38 mitogen-activated protein kinase (MAPK) signaling, providing ethnopharmacological relevance for its bioactive constituents in skin inflammatory disease [25]. Despite these established pharmacological profiles, the effects of AVC and SNG on TRPV3-associated calcium signaling and downstream inflammatory responses in keratinocytes have not been characterized.

In the present study, we initially screened approximately 1300 natural products by assessing NO production in carvacrol-stimulated human keratinocytes. Compounds showing an approximately 30% reduction in NO production relative to the corresponding carvacrol-stimulated control were preferentially retained as primary candidates. In addition, five compounds showing marked increases in NO production were preferentially retained for comparison, resulting in a total of 33 compounds for further evaluation. NO production and cell viability were assessed for these candidates, followed by secondary evaluation of TRPV3 and TSLP expression. AVC and SNG were subsequently selected for further mechanistic investigation based on the overall secondary screening profiles of TRPV3 and TSLP expression across the tested concentrations. Molecular docking, Fluo-3-based Ca^2+^ imaging, quantitative real-time PCR (qPCR), immunocytochemistry (ICC), and pathway inhibition assays were then used to characterize their predicted interactions with TRPV3 and their effects on TRPV3-associated inflammatory signaling.

## 2. Materials and Methods

### 2.1. Natural Product Library and Compounds

A library of 1300 natural compounds was obtained from the Korea Chemical Bank (KCB, Daejeon, Republic of Korea), comprising single-component natural products and natural product-like structures. Avicularin (AVC) and Senegenin (SNG) were selected as lead candidates based on primary screening and subsequent biological evaluation of TRPV3 and TSLP expression. Avicularin (AVC; Cat. No. PHL80361, ≥95% purity by HPLC) and senegenin (SNG; Cat. No. SML1001, ≥98% purity by HPLC) used in the validation experiments were purchased from Sigma-Aldrich (St. Louis, MO, USA).

All compounds were dissolved in dimethyl sulfoxide (DMSO) and stored at −20 °C prior to use. The final DMSO concentration in all assays did not exceed 0.1% (*v*/*v*). Carvacrol, used as a TRPV3 agonist; VIVIT peptide, used as an NFAT inhibitor; Bay 11-7082, used as an NF-κB inhibitor; and trpvicin (SML3758), used as a selective TRPV3 inhibitory control, were purchased from Sigma-Aldrich (St. Louis, MO, USA).

### 2.2. Cell Culture

HaCaT human keratinocytes (Cell Lines Service, Eppelheim, Germany; Cat. No. 300493; Cellosaurus: CVCL_0038) were cultured in RPMI-1640 medium, whereas HEK293 cells (American Type Culture Collection, Manassas, VA, USA; Cat. No. CRL-3216^TM^; Cellosaurus: CVCL_0063) were cultured in Dulbecco’s Modified Eagle Medium (DMEM). Both media were supplemented with 10% fetal bovine serum (FBS) and 1% penicillin–streptomycin at 37 °C in a humidified atmosphere containing 5% CO_2_.

### 2.3. Nitric Oxide (NO) Inhibition Assay

Anti-inflammatory activity was assessed using the Griess reagent assay for NO production. HaCaT cells were seeded in 96-well plates at a density of 5 × 10^4^ cells/well and allowed to attach overnight. Cells were pretreated with test compounds at concentrations of approximately 50 µM for 1 h, with the exact treatment concentration for each compound determined according to its respective stock concentration (Supplementary Table S1), and subsequently stimulated with carvacrol (100 µM) for 72 h. Culture supernatants were collected, and NO levels were measured by adding equal volumes of Griess reagent (1% sulfanilamide and 0.1% naphthylethylenediamine dihydrochloride in 5% phosphoric acid). Absorbance was read at 540 nm using a microplate reader. Cell viability was assessed in parallel using the 3-(4,5-dimethylthiazol-2-yl)-2,5-diphenyltetrazolium bromide (MTT) assay. MTT solution (5 mg/mL stock) was added to the culture medium at a 1:100 dilution and incubated for 2 h at 37 °C. The resulting formazan crystals were dissolved in DMSO, and absorbance was measured at 570 nm using a microplate reader. Cell viability was expressed relative to the vehicle-treated control.

The primary library screen was performed using a single well per compound. From approximately 1300 natural products, compounds showing an approximately 30% reduction in NO production relative to the corresponding carvacrol-stimulated control were preferentially retained as primary candidates. In addition, five compounds showing marked increases in NO production were preferentially retained for comparison, resulting in a total of 33 compounds for further evaluation. NO production and cell viability data for these 33 candidates are provided in Supplementary Table S1.

Based on NO production, cell viability, and chemical structure considerations, 11 compounds were prioritized for secondary evaluation. These compounds were evaluated at low and high concentrations for their effects on TRPV3 and TSLP expression by qRT-PCR in an exploratory screen performed once per condition (n = 1).

### 2.4. Molecular Docking Analysis

Molecular docking analysis was performed to predict the binding modes of AVC and SNG within TRPV3. The TRPV3 structures corresponding to the vanilloid binding site and S1–S4 site were obtained from the Protein Data Bank (PDB; accessed on 29 June 2026) using PDB IDs 8V6M and 8V6N, respectively [26]. Water molecules and non-polymer ligands were removed before receptor preparation. For the vanilloid site (PDB: 8V6M), the receptor was modeled using two pocket-lining subunits (chains A and B), incorporating 14 chain-A and 9 chain-B interfacial residues within 5 Å of the co-crystal ligand THCV. For the S1–S4 (voltage-sensor-like domain, VSLD) site (PDB: 8V6N), a single-subunit model (chain A, 14 residues) was used, as this pocket is lined entirely by one subunit. Polar hydrogens were added, and Gasteiger charges were assigned using OpenBabel 3.1.1.

The three-dimensional conformers of AVC (PubChem CID: 5490064) and SNG (PubChem CID: 12442762) were retrieved from PubChem. Docking simulations were conducted using AutoDock Vina v1.2.3. The grid centers were set at 108.53, 83.60, and 79.55 Å for the vanilloid site and 78.15, 70.08, and 118.94 Å for the S1–S4 site. The grid box size was set to 18 × 18 × 18 Å^3^, centered on the crystallographic ligand centroid of each binding site, with an exhaustiveness value of 32 and 20 docking modes generated per ligand. All docking calculations were performed in triplicate using three independent random seeds to assess pose reproducibility.

The top-ranked docking poses were visualized using PyMOL (The PyMOL Molecular Graphics System, version 2.5.0 Open-Source; Schrödinger, LLC, New York, NY, USA). Binding pocket surfaces were defined as receptor residues located within 5 Å of the bound ligands. Docking scores were reported in kcal/mol and interpreted qualitatively. Because AutoDock Vina is stochastic, minor variation in docking scores may occur depending on receptor and ligand preparation conditions.

To benchmark the docking protocol, cognate redocking was performed using the co-crystallized ligands from each structure: THCV (vanilloid site, PDB: 8V6M) and 2-APB (S1–S4 site, PDB: 8V6N). Grid centers were defined on the crystallographic ligand centroids (offset: 0.53 Å from THCV; 0.91 Å from 2-APB). Redocking was performed using the same protocol as compound screening (18 Å grid box, exhaustiveness 32, three independent seeds). Pose Root Mean Square Deviation (RMSD) was calculated against the crystallographic reference using symmetry-corrected heavy-atom RMSD (obrms). For 2-APB, a boron-to-carbon surrogate was used owing to the absence of boron parameters in AutoDock Vina; the crystallographic reference used the unmodified 2-APB structure. Docking scores are interpreted qualitatively rather than as quantitative potency rankings, consistent with the known limitations of empirical scoring functions at lipid-exposed binding sites.

### 2.5. Calcium Imaging

Calcium imaging was conducted based on the protocol reported previously [27]. Briefly, HEK293 cells were transiently transfected with a human TRPV3 cDNA in a pcDNA3.1 plasmid (2 µg per well) using FuGENE^®^ 4K Transfection Reagent (Promega, Madison, WI, USA). Cells were allowed to express TRPV3 for at least 24 h before calcium imaging. Functional expression of TRPV3 was assessed by the carvacrol-induced Ca^2+^-dependent fluorescence response. TRPV3-expressing HEK293 cells were then loaded with 5 µM Fluo-3/AM (Invitrogen, Eugene, OR, USA) and incubated at 37 °C for 40 min. After loading, cells were washed with 1× normal bath solution (NBS; 140 mM NaCl, 5 mM KCl, 2 mM CaCl_2_, 0.5 mM MgCl_2_, 10 mM glucose, and 5.5 mM HEPES, pH 7.4). Test compounds (50 µM) were applied 10 min prior to stimulation with carvacrol (300 µM). This concentration was selected based on established protocols for TRPV3 activation in heterologous overexpression systems, in which higher agonist concentrations are required to achieve robust and reproducible channel activation [3,5,28]. Trpvicin (200 µM), a selective TRPV3 inhibitor, was included as a positive inhibitory control [28]. Fluorescence signals were acquired using a fluorescence microscope (DMi8, Leica, Wetzlar, Germany) with excitation at 488 nm and emission at 515 nm. Time-lapse images were recorded for 3 min at 3-s intervals. Ca^2+^-dependent fluorescence responses were expressed as F/F_0_, where F represents the fluorescence intensity at a given time point and F_0_ represents the baseline fluorescence intensity at time zero. Image analysis was performed using ImageJ (NIH). The number of cells analyzed (n) represents the total number of cells obtained from at least three independent experiments. Data are presented as mean ± SEM from at least three independent experiments (3 wells per condition per experiment). Statistical comparisons are based on experiment-level means.

### 2.6. Quantitative Real-Time PCR (qRT-PCR)

For gene-expression analysis, HaCaT cells were pretreated with AVC, SNG, and/or the indicated pathway inhibitor, as appropriate, for 1 h, followed by stimulation with carvacrol for 3 h. Total RNA was extracted from treated HaCaT cells using TRIzol reagent (Invitrogen) according to the manufacturer’s protocol. cDNA was synthesized from 1 µg of total RNA using a reverse transcription kit. qRT-PCR was performed using SYBR Green master mix on a LightCycler^®^ 96 Real-Time PCR System (Roche Diagnostics, Mannheim, Germany). Gene expression was quantified using the 2^(−ΔΔCt) method with Glyceraldehyde-3-phosphate dehydrogenase (*GAPDH*) as the reference gene. The following primer sequences were used for qRT-PCR: *GAPDH* (forward: 5′-CTG GGC TAC ACT GAG CAC C-3′; reverse: 5′-AAG TGG TCG TTG AGG GCA ATG-3′), *TSLP* (forward: 5′-TAG CAA TCG GCC ACA TTG CC-3′; reverse: 5′-CTG AGT TTC CGA ATA GCC TG-3′), *TRPV3* (forward: 5′-TCC TCA CCT TTG TTC TCC TCC T-3′; reverse: 5′-CGC AAA CAC AGT CGG AAA TCA T-3′), and *TSLPR* (forward: 5′-GAG TGG CAG TCC AAA CAG GAA-3′; reverse: 5′-ACA TCC TCC ATA GCC TTC ACC-3′). For TRPV3 knockdown experiments, HaCaT cells were transfected with TRPV3 siRNA (sc-44170; Santa Cruz Biotechnology, Dallas, TX, USA) or Silencer™ Select Negative Control No. 1 siRNA (siCon; Cat. No. 4390843; Invitrogen, Thermo Fisher Scientific, Waltham, MA, USA) at a final concentration of 10 nM using Lipofectamine^®^ 2000 Transfection Reagent (Invitrogen, Thermo Fisher Scientific, Waltham, MA, USA) according to the manufacturer’s instructions. The siCon was used as a non-targeting siRNA control. Cells were stimulated with carvacrol 24 h after transfection. Knockdown efficiency was confirmed by qRT-PCR by comparing TRPV3 mRNA expression between siCon- and siTRPV3-transfected cells under the same stimulation conditions.

### 2.7. Immunocytochemistry (ICC)

HaCaT cells were seeded on coverslips in 8-well plates. For TRPV3 and TSLP expression analyses, cells were pretreated with AVC or SNG for 1 h, followed by carvacrol stimulation for 3 h before fixation. For signaling analyses, cells were pretreated with AVC, SNG, or the indicated pathway inhibitor for 1 h, followed by carvacrol stimulation for 5 min for phospho-NFATC2 (Ser54) or 20 min for phospho-NFκB p50 (Ser337) analysis. Following treatment, cells were fixed with 4% paraformaldehyde (PFA) for 30 min at room temperature, permeabilized with 0.1% Triton X-100 in phosphate-buffered saline (PBS) for 10 min, and blocked with 3% bovine serum albumin (BSA) in PBS for 1 h. Cells were incubated overnight at 4 °C with primary antibodies against TRPV3 (ab94582; Abcam, Cambridge, UK), TSLP (ab47943; Abcam, Cambridge, UK), phospho-NFATC2 (Ser54) (44-944G; Invitrogen, USA), and phospho-NFκB p50 (Ser337) (A-8; sc-271908; Santa Cruz Biotechnology, USA). Phospho-NFATC2 (Ser54) was assessed as an activation-associated phospho-epitope rather than as a direct marker of the canonical NFAT dephosphorylation event. After washing, cells were incubated with appropriate fluorophore-conjugated secondary antibodies for 1 h at room temperature. The following secondary antibodies were used: goat anti-mouse IgG H&L (Alexa Fluor^®^ 488; ab150117; Abcam, Cambridge, UK) and goat anti-rabbit IgG H&L (Alexa Fluor^®^ 488; ab150081; Abcam, Cambridge, UK).

Nuclei were counterstained with 4′,6-diamidino-2-phenylindole (DAPI). Images were acquired using a fluorescence microscope with a 20× objective and analyzed with ImageJ. For quantitative ICC analysis, one coverslip per condition was analyzed in each independent experiment, with at least 30 cells quantified per coverslip. The mean fluorescence intensity (MFI) across the analyzed cells was calculated to generate a single experiment-level value for statistical analysis. ICC analyses were performed in three independent experiments (n = 3). For signaling analyses, nuclear localization of phospho-NFATC2 (Ser54) and phospho-NFκB p50 (Ser337) was quantified using DAPI-defined nuclear regions.

### 2.8. Nuclear Factor Kappa B (NF-κB) and Nuclear Factor of Activated T Cells (NFAT) Inhibition Assays

To investigate the functional interplay between TRPV3 and downstream inflammatory signaling pathways, HaCaT cells were pretreated for 1 h with an NF-κB inhibitor, Bay 11-7082 (10 µM), the NFAT inhibitor, VIVIT peptide (10 µM), AVC (50 µM), or SNG (50 µM), either alone or in the indicated combinations, prior to carvacrol (100 µM) stimulation. Gene and protein expressions of inflammatory markers were subsequently assessed by qRT-PCR and ICC as described above.

### 2.9. Statistical Analysis

All data are expressed as mean ± standard error of the mean (SEM) from at least three independent experiments, unless otherwise stated. Quantitative immunocytochemistry (ICC) data are presented as mean ± standard deviation (SD). Independent experiments, rather than individual wells or cells, were considered the biological replicates and statistical units. For assays involving multiple wells or cells within an experiment, these measurements were averaged to obtain a single value for each independent experiment before statistical analysis. Statistical comparisons between groups were performed using one-way analysis of variance (ANOVA) followed by Tukey’s post hoc test. A *p*-value < 0.05 was considered statistically significant. All statistical analyses were conducted using GraphPad Prism (version 9.0).

## 3. Results

### 3.1. Identification of Candidate Compounds by Functional Screening and Molecular Docking

**Figure 1:**
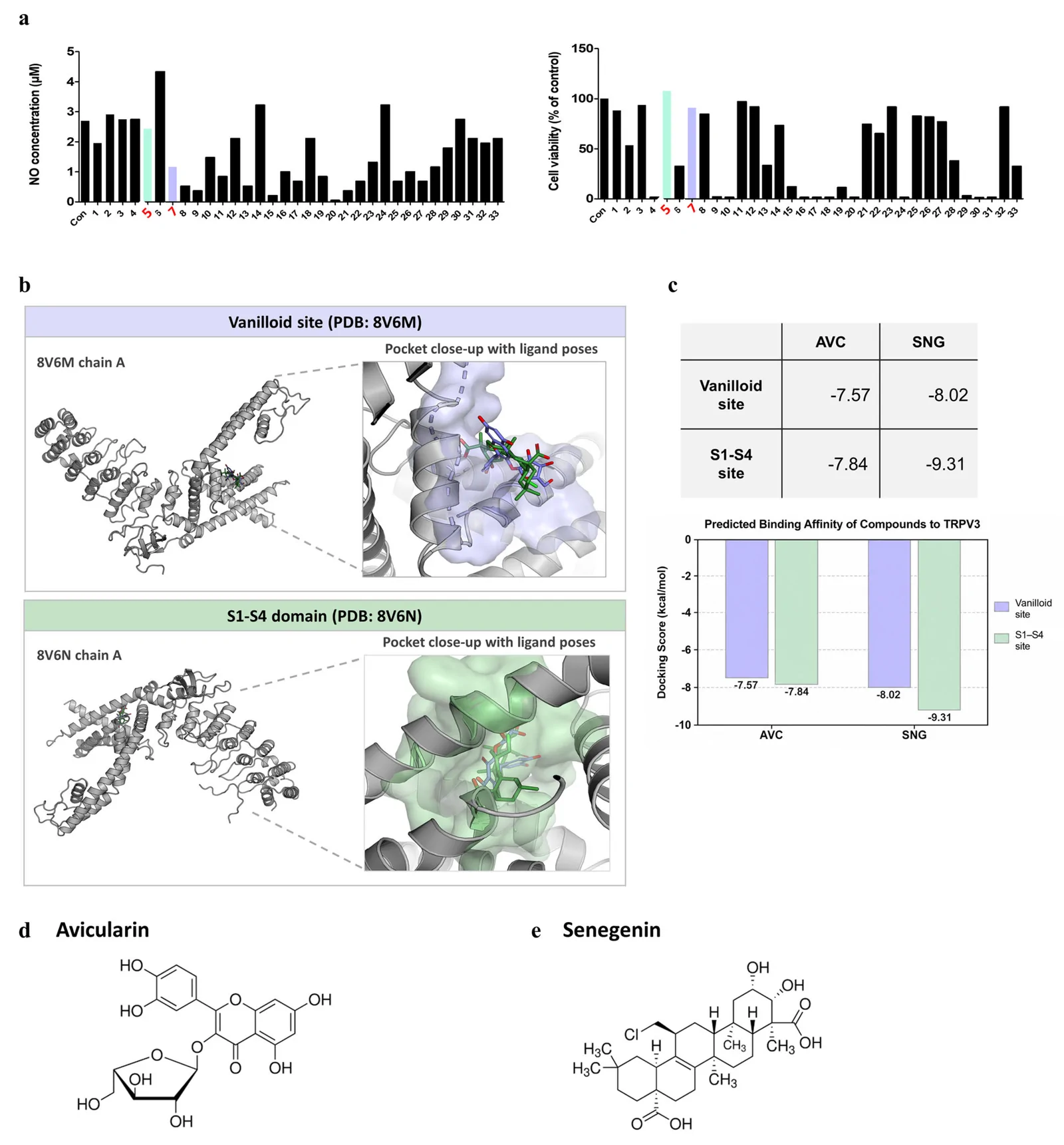
Functional screening and in silico characterization of AVC and SNG. (**a**) Nitric oxide (NO) production and cell viability profiles of the 33 candidate compounds retained from the initial screening of 1300-compound natural products. Compounds showing an approximately 30% reduction in NO production relative to the corresponding carvacrol-stimulated control were preferentially retained, together with five compounds showing marked increases in NO production for comparison. Avicularin (AVC, cyan) and senegenin (SNG, violet) are highlighted. (**b**) Structural visualization of AVC and SNG docked into the vanilloid binding site (PDB: 8V6M, upper panel) and the S1–S4 site (PDB: 8V6N, lower panel) of TRPV3. Left panels show the overall receptor topology, and right panels show close-up views of ligand poses within the binding pockets. (**c**) Predicted docking scores (kcal/mol) of AVC and SNG at the vanilloid site and S1–S4 site of TRPV3. (**d**,**e**) Chemical structures of AVC (**d**) and SNG (**e**).

To identify natural compounds capable of modulating TRPV3-associated inflammatory responses, a primary functional screen was conducted by measuring nitric oxide (NO) production in carvacrol-stimulated HaCaT keratinocytes treated with a 1300-compound natural product library. Compounds showing an approximately 30% reduction in NO production relative to the corresponding carvacrol-stimulated control were preferentially retained as primary candidates. In addition, five compounds showing marked increases in NO production were preferentially retained for comparison, resulting in a total of 33 compounds for further evaluation (Figure 1a and Supplementary Table S1). Cell viability was assessed in parallel using the 3-(4,5-dimethylthiazol-2-yl)-2,5-diphenyltetrazolium bromide (MTT) assay (Figure 1a). Based on their NO production, cell viability, and chemical structures, 11 of the 33 candidate compounds were prioritized for secondary evaluation. These 11 compounds were subsequently evaluated at low and high concentrations for their effects on TRPV3 and TSLP expression relative to the carvacrol-stimulated control. Based on the overall secondary screening profiles of TRPV3 and TSLP expression across the tested concentrations, AVC and SNG were selected for subsequent mechanistic investigation. Molecular docking was then performed to computationally characterize the potential interactions of AVC and SNG with two distinct TRPV3 structures representing the vanilloid binding site (PDB: 8V6M) and the S1–S4 site (PDB: 8V6N). Both avicularin (AVC) and senegenin (SNG) were predicted to occupy the ligand-binding pockets within TRPV3, with close-up structural views demonstrating predicted accommodation of each compound within the respective binding cavities (Figure 1b). Docking score analysis revealed favorable docking scores for both compounds at the two sites (Figure 1c). In the redocking-benchmarked docking protocol, AVC showed docking scores of −7.57 kcal/mol (vanilloid site) and −7.84 kcal/mol (S1–S4 site), and SNG showed docking scores of −8.02 kcal/mol (vanilloid site) and −9.31 kcal/mol (S1–S4 site; Supplementary Table S2). The top-ranked poses for both compounds were localized within the predefined docking pockets at both docking sites (centroid distances: AVC 1.8 Å at the vanilloid site and ≤2.1 Å at the S1–S4 site; SNG, 1.0 Å at the vanilloid site and ≤2.3 Å at the S1–S4 site).

Cognate redocking showed that a native-like THCV pose could be sampled within 0.56 Å RMSD (symmetry-corrected heavy-atom), and that of 2-APB within 1.5–2.2 Å RMSD (2 of 3 seeds < 2 Å; Supplementary Figure S1). The native-like pose was not consistently the top-ranked Vina output (top-ranked RMSD 3.6–6.3 Å for THCV; ~5.5 Å for 2-APB), which is expected for small, lipophilic ligands at a partially lipid-exposed site and is consistent with qualitative interpretation of docking scores. The top-ranked AVC and SNG poses localized within the intended pocket (centroid distance ≤ 2.3 Å from the co-crystal ligand) in all replicate runs at both sites, and their poses at the S1–S4 site overlap the crystallographic 2-APB position (Supplementary Figure S2 and Supplementary Table S2). The chemical structures of the two selected compounds are shown in Figure 1d,e.

### 3.2. Effects of AVC and SNG on Carvacrol-Induced Ca^2+^-Dependent Fluorescence Responses in TRPV3-Expressing Cells

**Figure 2:**
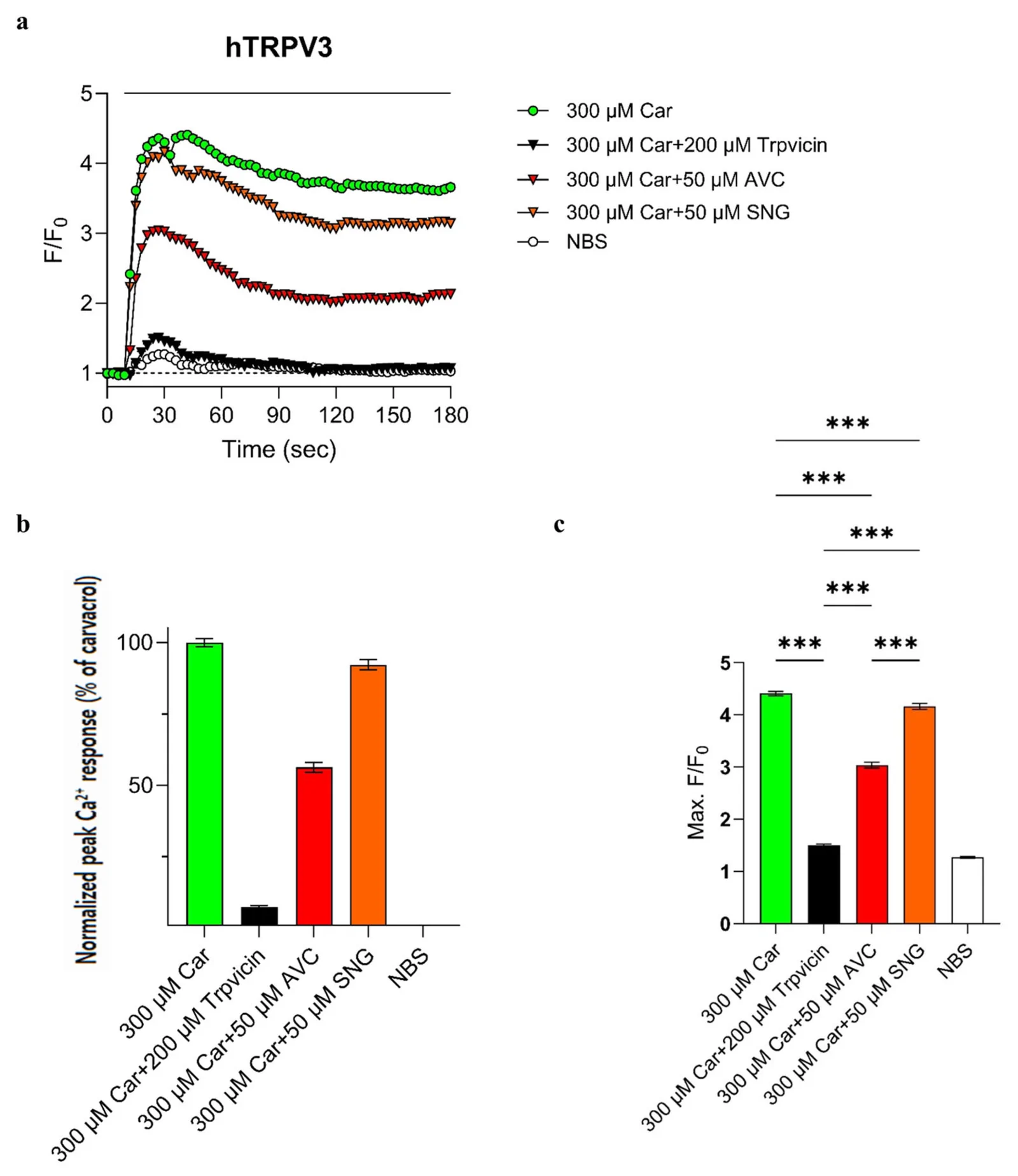
AVC and SNG attenuate carvacrol-induced Ca^2+^-dependent fluorescence responses in TRPV3-expressing cells. (**a**) Representative time-lapse fluorescence traces (F/F_0_) in TRPV3-expressing HEK293 cells stimulated with carvacrol (Car, 300 µM) in the presence of trpvicin (200 µM, a known TRPV3 inhibitor), AVC, SNG, or vehicle (NBS). (**b**) Quantification of peak Ca^2+^-dependent fluorescence responses normalized to the carvacrol-treated group, which was set to 100%. (**c**) Quantification of the actual maximum F/F_0_ values across treatment groups. Data are presented as mean ± SEM from at least three independent experiments. (3 wells per condition per experiment). *** *p* < 0.001.

To evaluate the effect of AVC and SNG on carvacrol-induced Ca^2+^-dependent fluorescence responses, Ca^2+^-dependent fluorescence was measured using Fluo-3-based calcium imaging in TRPV3-expressing HEK293 cells. Stimulation with carvacrol (300 µM) induced a rapid and sustained increase in Ca^2+^-dependent fluorescence (F/F_0_) (Figure 2a). This response was markedly attenuated by trpvicin (200 µM), a known TRPV3 inhibitor, which was included as a positive inhibitory control.

Pretreatment with AVC (50 µM) or SNG (50 µM) significantly attenuated the carvacrol-induced Ca^2+^-dependent fluorescence responses compared with the carvacrol-stimulated control, as evidenced by reduced fluorescence intensity over the 180-s recording period. Quantitative analysis of peak F/F_0_ values confirmed that both compounds significantly decreased the normalized peak responses (Figure 2b), and maximum F/F_0_ values were likewise significantly reduced (Figure 2c). Notably, AVC produced a greater attenuation of Ca^2+^-dependent fluorescence response than SNG under the tested conditions.

To further characterize the concentration dependence of these effects, additional concentration–response experiments were performed. Both AVC and SNG showed concentration-dependent attenuation of carvacrol-induced Ca^2+^-dependent fluorescence responses, with approximate apparent IC_50_ estimates of 200 µM for AVC and 100 µM for SNG (Supplementary Figure S3). These estimates should be interpreted cautiously because the tested concentration range did not adequately define both plateaus of the concentration–response curves.

### 3.3. AVC and SNG Attenuate TRPV3-Associated TSLP Signaling

Having shown that AVC and SNG attenuate carvacrol-induced Ca^2+^-dependent fluorescence responses in TRPV3-expressing cells, we next investigated whether these compounds regulate TRPV3 expression and its downstream inflammatory signaling. The mRNA expression levels of *TRPV3*, *TSLP*, and *TSLPR* were analyzed following carvacrol stimulation.

**Figure 3:**
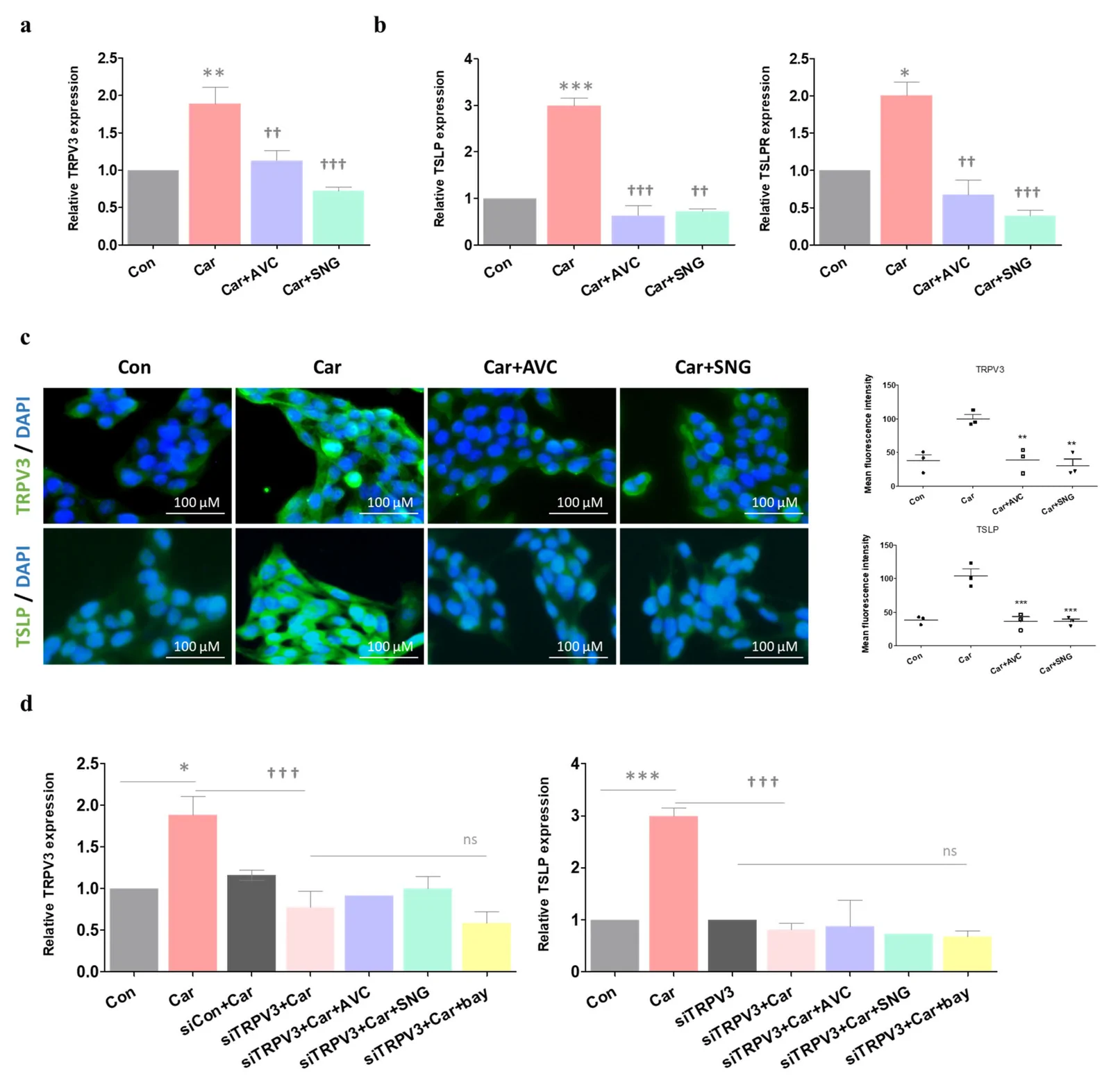
AVC and SNG attenuate TRPV3 expression and downstream TSLP signaling. (**a**) Relative mRNA expression of *TRPV3*, (**b**) *TSLP* (left) and *TSLPR* (right) in HaCaT cells treated with carvacrol (Car) alone or in combination with AVC or SNG, as assessed by qRT-PCR. (**c**) Representative immunocytochemical (ICC) images showing TRPV3 (upper, green) and TSLP (lower, green) protein expression counterstained with nuclear staining (DAPI, blue). For quantitative analysis, one coverslip per condition was analyzed in each independent experiment, with at least 30 cells quantified per coverslip. The mean MFI from each independent experiment was used as the statistical unit. Data are presented as mean ± SD from three independent experiments (n = 3), with individual experiment-level values shown. Scale bar = 100 µm. (**d**) Relative mRNA expression of TRPV3 (left) and TSLP (right) under siRNA-mediated TRPV3 knockdown conditions. A non-targeting siRNA control (siCon) was included to validate TRPV3-specific silencing under carvacrol stimulation. The effects of AVC, SNG, or Bay 11-7082 were further evaluated in siTRPV3-transfected, carvacrol-stimulated cells. Data are presented as mean ± SEM. Statistical significance was determined relative to the carvacrol-treated group: * denotes comparison between the control and carvacrol-treated groups (* *p* < 0.05, ** *p* < 0.01, *** *p* < 0.001; † denotes comparison between the siCon + carvacrol and siTRPV3 + carvacrol groups for TRPV3, and between the carvacrol and siTRPV3 + carvacrol groups for TSLP (†† *p* < 0.01, ††† *p* < 0.001); ns, not significant). Scale bars = 100 µm. Images were acquired using a 20× objective.

As shown in Figure 3a,b, carvacrol stimulation significantly upregulated the mRNA expression of *TRPV3*, *TSLP*, and *TSLPR* relative to the unstimulated control. Treatment with AVC or SNG markedly reduced the expression of all three genes, consistent with transcriptional modulation of the TRPV3-TSLP/TSLPR axis.

At the protein level, ICC confirmed carvacrol-induced upregulation of TRPV3 and TSLP, both of which were substantially reduced by AVC or SNG treatment (Figure 3c), consistent with the qRT-PCR results.

To confirm the involvement of TRPV3, siRNA-mediated TRPV3 knockdown (siTRPV3) was performed. Effective TRPV3 knockdown was confirmed by qRT-PCR using a non-targeting siRNA control (siCon). Under carvacrol-stimulated conditions, TRPV3 mRNA expression was reduced by approximately 79% in siTRPV3-transfected cells compared with siCon-transfected cells (Supplementary Figure S4). This knockdown-validation experiment was performed independently of the mechanistic experiment shown in Figure 3d; accordingly, the magnitude of TRPV3 knockdown may differ between the two datasets due to experimental variability, although both experiments confirmed significant TRPV3 silencing under carvacrol stimulation. Under carvacrol stimulation, siTRPV3 transfection significantly attenuated carvacrol-induced *TRPV3* and *TSLP* expression compared with the carvacrol-treated group. Furthermore, additional treatment with AVC, SNG, or Bay 11-7082 under siTRPV3-transfected conditions did not further suppress *TRPV3* or *TSLP* expression (ns), supporting the involvement of TRPV3 in the observed effects of AVC and SNG (Figure 3d).

### 3.4. AVC and SNG Attenuate TRPV3-Associated NFAT/NF-κB Signaling and Downstream TSLP Expression

**Figure 4:**
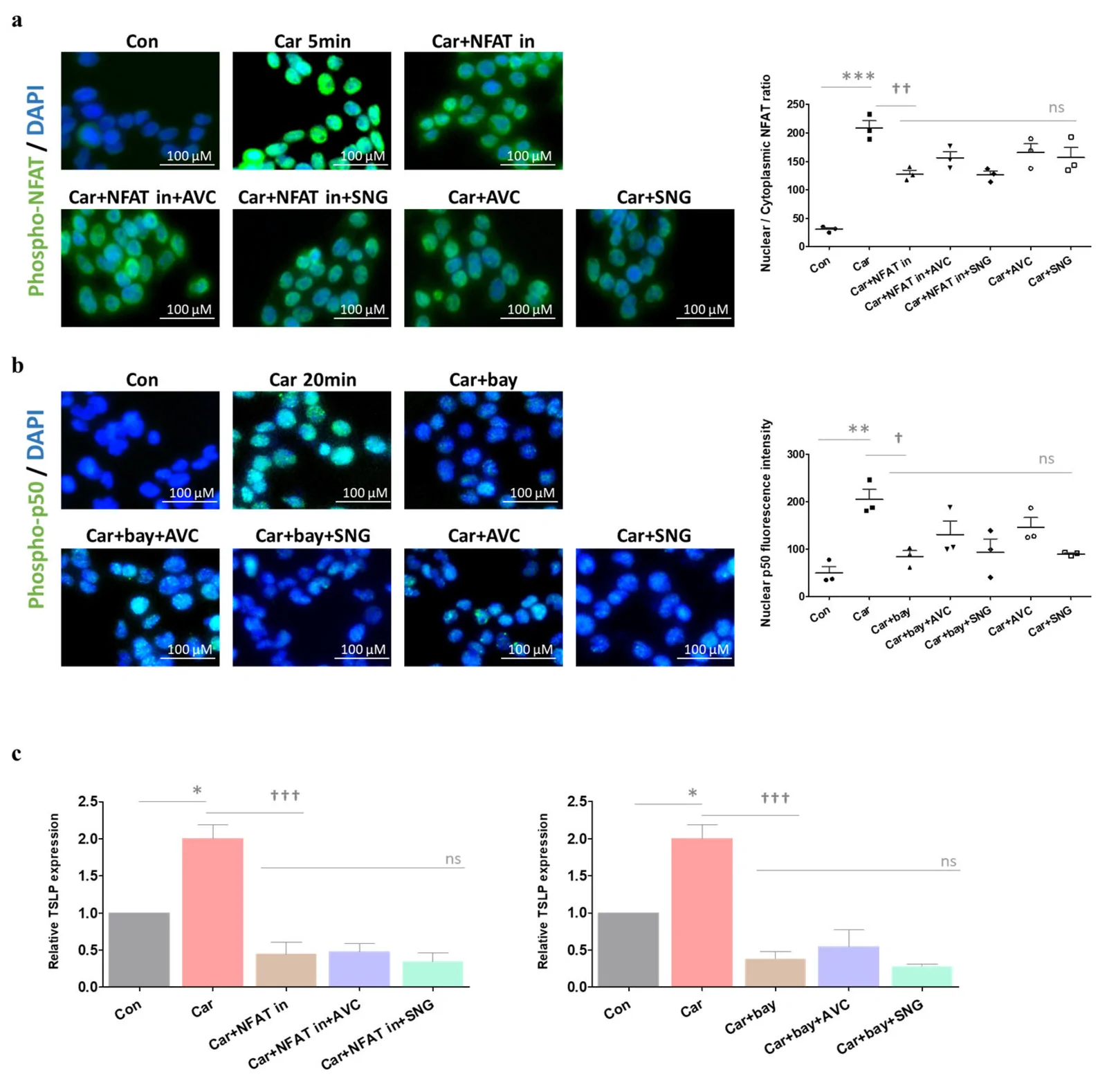
AVC and SNG attenuate TRPV3-associated NFAT/NF-κB signaling and downstream TSLP expression. (**a**) Representative ICC images and quantification of nuclear/cytoplasmic phospho-NFATC2 (Ser54) fluorescence following carvacrol stimulation with or without NFAT inhibitor, AVC, or SNG. (**b**) Representative ICC images and quantification of nuclear phospho-p50 (Ser337) fluorescence following carvacrol stimulation (20 min) with or without Bay 11-7082, AVC, or SNG. For ICC quantification, ≥30 cells from one coverslip per condition were analyzed per experiment, with the experiment-level mean used for statistical analysis (n = 3). Data are presented as mean ± SD with individual biological replicate values. Scale bar = 100 µm. (**c**) qRT-PCR analysis of *TSLP* mRNA expression in cells co-treated with NFAT inhibitor + AVC/SNG (left) or Bay 11-7082 + AVC/SNG (right). Data are presented as mean ± SEM from at least three independent experiments. * *p* < 0.05, ** *p* < 0.01, *** *p* < 0.001; † *p* < 0.05, †† *p* < 0.01, ††† *p* < 0.001; ns, not significant. Scale bars = 100 µm. Images were acquired using a 20× objective.

TRPV3-mediated Ca^2+^ influx has been reported to activate NFAT and NF-κB signaling, driving downstream TSLP expression in keratinocytes [13]. To examine whether AVC and SNG modulate TRPV3-downstream signaling pathways, the nuclear localization of phospho-NFATC2 (Ser54) and phospho-p50 (Ser337) was assessed by ICC. Carvacrol stimulation (5 min) significantly increased the nuclear/cytoplasmic phospho-NFAT ratio compared with the unstimulated control (Figure 4a). AVC or SNG reduced nuclear phospho-NFATC2 (Ser54) to levels comparable to those observed with the NFAT inhibitor (ns vs. NFAT inhibitor), and co-treatment with AVC or SNG in the presence of the NFAT inhibitor produced no additional reduction.

Similarly, carvacrol stimulation (20 min) significantly elevated nuclear phospho-p50 (Ser337) fluorescence intensity (Figure 4b). AVC and SNG reduced nuclear p50 accumulation to levels comparable to Bay 11-7082 (ns vs. Bay 11-7082), and combination treatment with Bay 11-7082 did not result in further suppression.

Carvacrol-induced *TSLP* mRNA expression was significantly suppressed by the NFAT inhibitor or Bay 11-7082 alone (Figure 4c). Co-treatment with AVC or SNG in the presence of either inhibitor did not further reduce *TSLP* expression (ns), consistent with convergence of their effects on NFAT- and NF-κB–associated signaling.

## 4. Discussion

In the present study, we employed an integrated approach combining NO inhibition screening, molecular docking, and multi-level in vitro validation to identify natural compounds capable of modulating TRPV3-associated inflammatory signaling in keratinocytes. From a 1300-compound natural product library, AVC and SNG were identified as candidate modulators of the TRPV3–TSLP axis. Both compounds demonstrated favorable docking scores at the TRPV3 S1–S4 domain, attenuation of carvacrol-induced Ca^2+^-dependent fluorescence responses, and reduced TRPV3 and TSLP expression at both mRNA and protein levels. Mechanistically, AVC and SNG attenuated the nuclear translocation of phospho-NFATC2 and phospho-p50, with no additional suppression observed upon co-treatment with selective pathway inhibitors, consistent with convergence of their effects on TRPV3-associated NFAT/NF-κB signaling. Because NO production is not a TRPV3-specific endpoint, the primary screening strategy may also identify compounds with broader anti-inflammatory or antioxidant activities. Therefore, the NO assay was used as an initial functional screening tool, whereas subsequent TRPV3/TSLP expression analyses and Ca^2+^-dependent fluorescence measurements were used to further evaluate TRPV3-associated effects.

TRPV3 has gained increasing attention as a therapeutic target in inflammatory skin disease. Gain-of-function mutations in TRPV3 are causally linked to Olmsted syndrome [11], a rare genodermatosis characterized by severe palmoplantar keratoderma and pruritus, supporting the pathological relevance of TRPV3 hyperactivity in skin. In AD, *TRPV3* expression is elevated in both lesional and non-lesional epidermis, and channel activation correlates with itch severity and skin barrier impairment [12]. The downstream upregulation of TSLP by TRPV3 activity represents a critical mechanistic link between thermal and chemical stimuli and type 2 inflammatory responses [13,15]. Importantly, TRPV3 activation in keratinocytes has been directly shown to trigger NF-κB pathway activation in a Ca^2+^-dependent manner [8], providing a mechanistic basis for the pathway-level findings observed in the present study.

AVC is a flavonoid glycoside with established anti-inflammatory properties, including reduction of IL-1β, IL-6, IL-8, and MMPs in TNF-α-stimulated synovial cells [22,29]. However, to our knowledge, this is the first study to report modulation of TRPV3-associated signaling by AVC and SNG. The docking scores of AVC at the vanilloid site (−7.57 kcal/mol) and S1–S4 site (−7.84 kcal/mol) suggest predicted interaction within putative ligand-binding regions of TRPV3. The multiple hydroxyl groups of the flavonoid glycoside scaffold may facilitate predicted hydrogen-bond interactions with polar residues lining the binding pocket, as suggested by the docking analysis (Figure 1b). Notably, flavonoids have been reported to modulate TRP channel activity; apigenin, a structurally related flavone, was shown to activate TRPV4 [30], supporting the broader concept that flavonoid scaffolds can modulate members of the TRPV channel family.

SNG is a pentacyclic triterpenoid sapogenin derived from *Polygala tenuifolia* and has been reported to possess anti-inflammatory and neuroprotective activities through modulation of NF-κB/NLRP3 signaling [24,31]. In addition, extracts of *P. tenuifolia* have shown beneficial effects in experimental atopy-like dermatitis models [25]. The favorable docking scores of SNG at the vanilloid site (−8.02 kcal/mol) and S1–S4 site (−9.31 kcal/mol) in the redocking-benchmarked docking protocol are consistent with extensive hydrophobic and van der Waals interactions between the bulky triterpenoid scaffold and the hydrophobic transmembrane binding pocket of TRPV3. These scores are interpreted qualitatively, as the raw score difference between SNG and AVC falls within AutoDock Vina’s reported standard error, and ligand efficiency normalization yields nearly identical values for both compounds (Supplementary Table S3). To our knowledge, modulation of TRPV3-associated Ca^2+^ responses by SNG has not been previously reported. It should be noted that AVC, as a flavonoid glycoside, may interact with targets beyond TRPV3, including other TRP channel family members such as TRPV1, TRPV4, and TRPA1. Although the present findings support involvement of TRPV3, the possibility of interactions with other TRP channels, including TRPV1, TRPA1, and TRPV4, cannot be excluded. Future electrophysiological studies comparing representative TRP channels will be necessary to establish the selectivity profile of AVC and SNG. In the absence of formal cross-TRP selectivity data, the observed anti-inflammatory effects cannot be attributed exclusively to TRPV3 channel modulation. The siRNA knockdown data presented here (Figure 3d) support the involvement of TRPV3 in the keratinocyte system, but selectivity profiling against representative TRP channels remains an important direction for future validation.

These findings support compatibility of AVC- and SNG-mediated effects with TRPV3-associated NFAT/NF-κB signaling, while not excluding potential direct or TRPV3-independent effects on these downstream pathways. TRPV3-mediated Ca^2+^ influx activates calcineurin/NFAT and NF-κB cascades, which cooperatively drive *TSLP* transcription [13,32]. Although NFAT nuclear translocation is classically regulated by calcineurin-mediated dephosphorylation, inducible phosphorylation within the NFATC2 transactivation domain has been associated with transcriptional activity [33]. Accordingly, phospho-NFATC2 (Ser54) was used in the present study as an activation-associated phospho-epitope, rather than as a direct measure of the canonical calcineurin-mediated NFAT dephosphorylation event [33]. Although TSLPR expression in keratinocytes has been demonstrated in our previous study using both NHEK and HaCaT cells [13], its functional significance in keratinocytes remains unclear, and TSLPR expression alone does not establish an autocrine TSLP–TSLPR amplification loop. The siRNA knockdown data further support the involvement of TRPV3 in the observed effects of AVC and SNG. Together, these findings suggest that AVC and SNG modulate TRPV3-associated inflammatory signaling; however, TRPV3-independent effects on downstream pathways cannot be excluded. Notably, AVC and SNG also reduced TRPV3 mRNA and protein expression, suggesting regulation of TRPV3 at both functional and expression levels. However, whether the attenuation of carvacrol-induced Ca^2+^-dependent fluorescence responses and reduction in TRPV3 expression share a common mechanism or represent distinct actions remains to be determined.

Several limitations of the present study should be acknowledged. First, the in vitro models used in this study do not fully recapitulate the complexity of cutaneous inflammation involving interactions among keratinocytes, immune cells, and sensory neurons. Validation in primary human keratinocytes, three-dimensional skin equivalents, and in vivo models will therefore be important. Second, the calcium-imaging experiments did not include mock-transfected or empty-vector HEK293 controls or AVC- and SNG-alone controls. Therefore, although AVC and SNG attenuated carvacrol-induced Ca^2+^-dependent fluorescence responses in TRPV3-expressing HEK293 cells, these findings do not establish direct inhibition of TRPV3 or exclude potential effects on endogenous Ca^2+^-handling mechanisms. The TRPV3 knockdown data support the involvement of TRPV3 in the observed cellular responses; however, comparative electrophysiological profiling against representative TRP channels, including TRPV1, TRPA1, and TRPV4, as well as additional control experiments, will be necessary to further characterize TRPV3 involvement and establish target selectivity [3,5,28]. Third, molecular docking provides only computational predictions of ligand binding, and future biophysical studies are warranted to validate these interactions. In addition, although the initial mechanistic validation was performed at a single screening concentration (50 µM), subsequent concentration–response analysis demonstrated concentration-dependent attenuation of carvacrol-induced Ca^2+^-dependent fluorescence responses in TRPV3-expressing cells. Although SNG showed a lower approximate apparent IC_50_ estimate than AVC, this should not be interpreted as indicating greater overall inhibitory efficacy, because potency and maximal efficacy represent distinct pharmacological properties. Moreover, because the tested concentration range did not adequately define both plateaus of the concentration–response curves, these apparent IC_50_ estimates should be interpreted cautiously. Nevertheless, further pharmacological characterization, including direct electrophysiological measurements and assessment of potential off-target effects, will be required. Finally, whether the effective in vitro concentration (50 µM) of AVC and SNG can be achieved in the skin following topical application remains unclear, warranting further investigation of their skin permeability, solubility, and formulation strategies for local delivery. Future studies using established pruritic dermatitis models, including the imiquimod-induced mouse model [34], are warranted to evaluate the effects of topical AVC/SNG application on pruritic behavior, skin lesion pathology, and cutaneous TSLP expression.

Beyond its mechanistic significance, the present findings may provide a basis for further investigation of AVC and SNG in inflammatory skin disorders. Aberrant epithelial-derived TSLP production is recognized as a key driver of type 2 inflammation not only in AD but also in chronic prurigo and other chronic inflammatory skin disorders. Therefore, modulation of TRPV3-associated TSLP signaling by naturally derived compounds such as AVC and SNG may provide a rationale for further investigation of TRPV3-targeted approaches in TSLP-associated inflammatory skin disorders.

## 5. Conclusions

This study employed an integrated in silico and in vitro approach to identify AVC, a flavonoid constituent of the ethnomedicinally relevant plant *Polygonum aviculare* L., and SNG, the key bioactive aglycone of *Polygalae Radix* (*Polygala tenuifolia*), as natural modulators of TRPV3-associated inflammatory signaling in human keratinocytes. Both compounds demonstrated favorable docking scores and predicted docking poses within the TRPV3 S1–S4 domain, attenuated carvacrol-induced Ca^2+^-dependent fluorescence responses, reduced TRPV3, TSLP, and TSLPR mRNA expression, and reduced TRPV3 and TSLP protein expression. Mechanistically, AVC and SNG attenuated NFAT and NF-κB nuclear translocation, with pathway inhibition producing no additive effect, consistent with convergence of their effects on TRPV3-associated NFAT/NF-κB–TSLP signaling. Collectively, these findings support AVC and SNG as candidate compounds for further investigation of TRPV3-associated inflammatory pathways in pruritic skin disorders.

## Data Availability

The data supporting this study’s findings are available from the corresponding author upon reasonable request.

## Supplementary Materials

The following supporting information can be downloaded at: https://www.mdpi.com/article/10.3390/cells15181659/s1, Supplementary Figure S1: Cognate redocking samples the native THCV pose (vanilloid site, PDB: 8V6M). Supplementary Figure S2: Top-ranked AVC/SNG poses co-localize with crystallographic 2-APB (S1–S4/VSLD, PDB: 8V6N). Supplementary Figure S3: Concentration–response effects of AVC and SNG on carvacrol-induced Ca^2+^-dependent fluorescence responses in TRPV3-expressing HEK293 cells. Supplementary Figure S4: Validation of siRNA-mediated TRPV3 knockdown in HaCaT cells. Supplementary Table S1: Primary screening data and secondary evaluation status of candidate compounds identified from the natural product library. Supplementary Table S2: Comparison of TRPV3 docking scores obtained using the original and redocking-benchmarked protocols (kcal/mol). Supplementary Table S3: Docking protocol validation and supporting metrics.

## Abbreviations

The following abbreviations are used in this manuscript:

AD	Atopic dermatitis
ANOVA	Analysis of variance
AVC	Avicularin
BSA	Bovine serum albumin
Ca^2+^	Calcium
COX-2	Cyclooxygenase-2
DAPI	4′,6-Diamidino-2-phenylindole
DMEM	Dulbecco’s Modified Eagle Medium
DMSO	Dimethyl sulfoxide
GAPDH	Glyceraldehyde-3-phosphate dehydrogenase
ICC	Immunocytochemistry
IL	Interleukin
iNOS	Inducible nitric oxide synthase
MAPK	Mitogen-activated protein kinase
MFI	Mean fluorescence intensity
MMP	Matrix metalloproteinase
MTT	3-(4,5-Dimethylthiazol-2-yl)-2,5-diphenyltetrazolium bromide
NFAT	Nuclear factor of activated T cells
NF-κB	Nuclear factor kappa B
NO	Nitric oxide
PBS	Phosphate-buffered saline
PDB	Protein Data Bank
PFA	Paraformaldehyde
PGE_2_	Prostaglandin E_2_
PKA	Protein kinase A
qRT-PCR	Quantitative real-time polymerase chain reaction
RMSD	Root mean square deviation
SEM	Standard error of the mean
SNG	Senegenin
THCV	Tetrahydrocannabivarin
TSLP	Thymic stromal lymphopoietin
TSLPR	Thymic stromal lymphopoietin receptor
TRP	Transient receptor potential
TRPV3	Transient receptor potential vanilloid 3
VSLD	Voltage-sensor-like domain
