# Supplementary material for: Avicularin and Senegenin Suppress TRPV3-Associated TSLP Expression in Human Keratinocytes
Source: Cells. 2026 Sep 14;15(18):1659. doi: 10.3390/cells15181659 (PMC13605539; doi:10.3390/cells15181659)
Supplement: Supplementary file 1 [file cells-15-01659-s001.zip › cells-4498815-supplementary.pdf]

Supplementary Table S1. Primary screening data and secondary evaluation status of candidate compounds identified from the natural product library.

Library ID	Treatment concentration (μM)	NO production (% of control)	Cell viability (% of control)	TSLP (% of Car control)	TRPV3 (% of Car control)
27 D04	52.5	72.5	88.14154	70.2	ND
27 E09	49.4	107.9	53.57508		
28 C03	50.6	102	93.58093	16.4	0.14
28 D03	50.9	102	1.579178		
**SNG**	50	90.2	107.6181	42.9	10.4
28 D07	51.3	160.9	32.54862		
**AVC**	55	43	90.86124	34.2	6
28 A10	52.5	19.4	84.51528	120.6	63.3
30 C07	49.9	13.6	1.959351		
30 E08	51.2	54.8	1.549934		
30 E09	49	31.2	97.03173	18.3	12.5
30 E10	48.1	78.4	91.97251	69.3	16.9
32 D04	48.8	19.4	33.33821		
32 E04	53.4	119.6	73.22708		
32 D06	47	7.7	11.99006		
32 E08	51.8	37.1	1.491446		
33 D05	49.9	25.3	1.52069		
33 G07	100	78.4	1.608422		
33 E08	49.9	31.2	11.22971		
34 D04	52	1.8	1.579178		
34 D05	50	13.6	74.51382		
34 E08	50	25.3	65.12648		
35 E08	51	48.9	91.68007	175.3	12
35 D10	49.9	119.6	1.579178		
36 D09	51	25.3	82.58517	55.1	27.9
36 E09	51	37.1	81.85407	52.1	14.7
37 D06	49.9	25.3	76.94107	139.5	ND
37 E08	54.9	43	37.92952		
38 D04	51.4	66.6	3.187601		
39 H05	46.1	102	1.257494		
42 E10	48.8	78.4	1.66691		
43 C07	50.4	72.5	91.65083		
43 A08	51.5	78.4	32.54862		

Note: NO production was normalized to the carvacrol-stimulated control, which was set to 100%. Values below 100% indicate reduced NO production relative to the carvacrol-stimulated control, whereas values above 100% indicate increased NO production. Secondary qRT-PCR screening of

the 11 prioritized compounds was exploratory and performed once per condition (n = 1). Where a valid measurement was not available, the value is reported as ND (not determined). The exact treatment concentrations varied slightly among compounds according to their respective stock concentrations.

Supplementary Table S2. Comparison of TRPV3 docking scores obtained using the original and redocking-benchmarked protocols (kcal/mol).

Compound	Site	Original (24 Å box, chain A)	Redocking-benchmarked (18 Å box, pocket subunits)	Range over 3 seeds	Pose vs co-crystal ligand
Avicularin (AVC)	Vanilloid (8V6M)	−5.94	−7.57	−7.39 – −7.77	in-pocket, 1.8 Å
	S1–S4 / VSLD (8V6N)	−7.49	−7.84	−7.74 – −7.99	in-pocket, ≤2.1 Å
Senegenin (SNG)	Vanilloid (8V6M)	−6.92	−8.02	−7.94 – −8.10	in-pocket, 1.0 Å
	S1–S4 / VSLD (8V6N)	−9.36	−9.31	−9.13 – −9.64	in-pocket, ≤2.3 Å

Note: AutoDock Vina v1.2.3, exhaustiveness 32, mean of 3 independent runs (different seeds). Receptor/ligand prepared with Open Babel 3.1.1. Vanilloid site (8V6M) is interfacial and was modeled with the two pocket-lining subunits; the intrasubunit S1–S4 site (8V6N) was modeled with a single subunit. Revised values represent the top-ranked poses localized within the predefined docking pockets ('in-pocket' = centroid distance to the crystallographic co-crystal ligand: THCV for vanilloid, 2-APB for S1–S4) in all runs. Scores are interpreted qualitatively (see ***Supplementary Table S3****).*

Supplementary Table S3. Docking protocol validation and supporting metrics.

Metric	Value
THCV cognate redock — native-like pose sampled (vanilloid, 8V6M)	0.56 Å (ranks 3–4 of 20 output poses)
THCV cognate redock — top-ranked (rank-1) pose RMSD	3.6 – 6.3 Å<br>(native-like pose not top-ranked)
2-APB (B→C surrogate) cognate redock — native-like pose sampled (S1–S4, 8V6N)	1.5–2.2 Å (ranks 14–20; < 2 Å in 2/3 seeds)
2-APB (B→C surrogate) — top-ranked (rank-1) pose RMSD	~5.5 Å (native-like pose not top-ranked)
AVC / SNG top-ranked pose location (all runs)	in-pocket,<br>≤ 2.3 Å of co-crystal ligand
Grid center vs. crystallographic ligand	0.53 Å (THCV); 0.91 Å (2-APB)
Vanilloid pocket residues (chain A / chain B)	14 / 9 (interfacial)
S1–S4/VSLD pocket residues (chain A / other)	14 / 0 (intrasubunit)
AVC / SNG heavy-atom count	31 / 37
AVC / SNG ligand efficiency at S1–S4 (kcal/mol/atom)	−0.253 / −0.252 (nearly identical)
SNG−AVC score gap (S1–S4) vs. Vina standard error	1.47 vs 2.85 kcal/mol (gap < error)

Note: Cognate redocking SAMPLES the experimentally observed pose but does NOT consistently rank it first for the small co-crystal ligands; for the larger AVC/SNG, the top-ranked pose lies in-pocket. Docking scores are therefore interpreted qualitatively, not as quantitative potency rankings. RMSD symmetry-corrected (obrms). 2-APB modeled with boron->carbon (AutoDock Vina lacks boron parameters). Vina standard error from Trott and Olson, J Comput Chem 2010. Nearly identical ligand efficiencies indicate the SNG-AVC raw-score difference reflects ligand size, not binding strength.

Supplementary Figure Legends

Supplementary Figure S1. Cognate redocking samples the native THCV pose (vanilloid site, PDB: 8V6M).

Cognate redocking samples the native THCV pose at the vanilloid site (PDB: 8V6M). Crystallographic THCV (green) overlaid with a native-like redocked pose sampled by the validated protocol (cyan; heavy-atom RMSD 0.56 Å, rank 3 of 20 sampled, not top-ranked). Pocket residues from chain A (wheat) and the adjacent chain B (orange) illustrate the subunit interface.

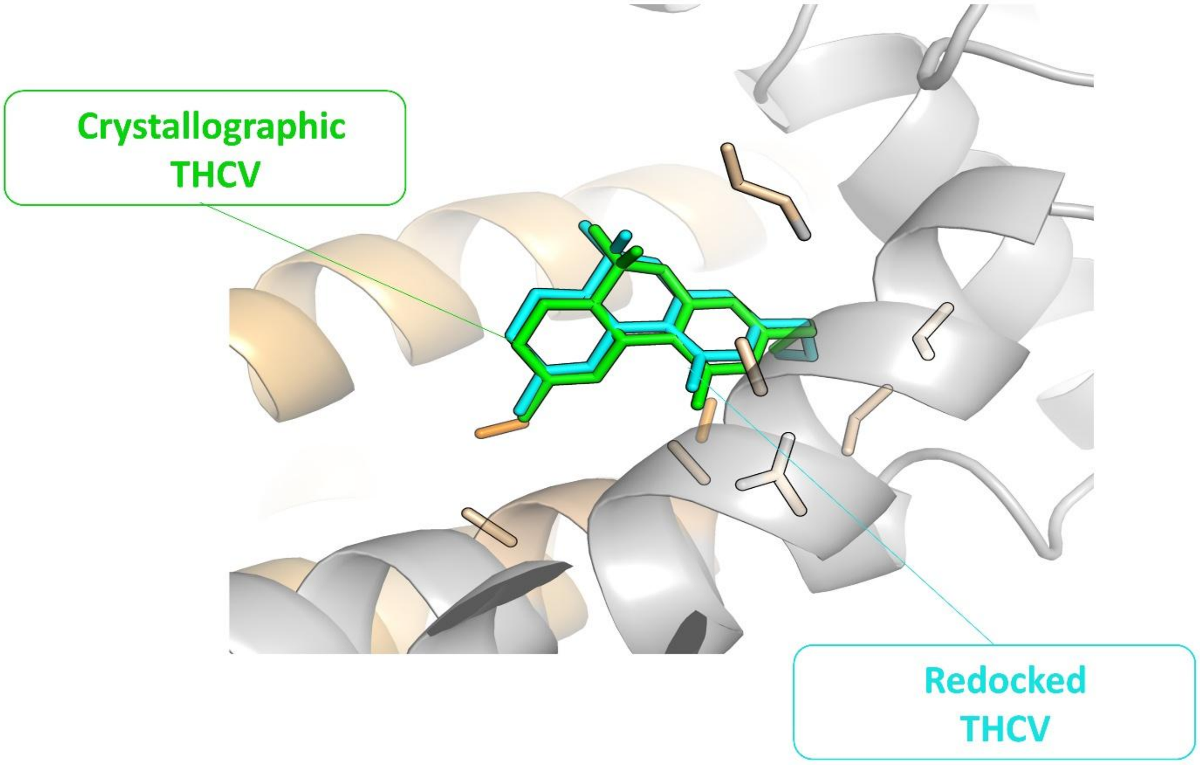

Supplementary Figure S2. Top-ranked AVC/SNG poses co-localize with crystallographic 2-APB (S1–S4/VSLD, PDB: 8V6N).

Top-ranked AVC and SNG poses co-localize with crystallographic 2-APB at the S1–S4/VSLD site (PDB: 8V6N). Top-ranked docked AVC (cyan) and SNG (magenta) overlap the crystallographic 2-APB pose (yellow) within 2.1–2.3 Å, supporting a predicted interaction with the VSLD pocket.

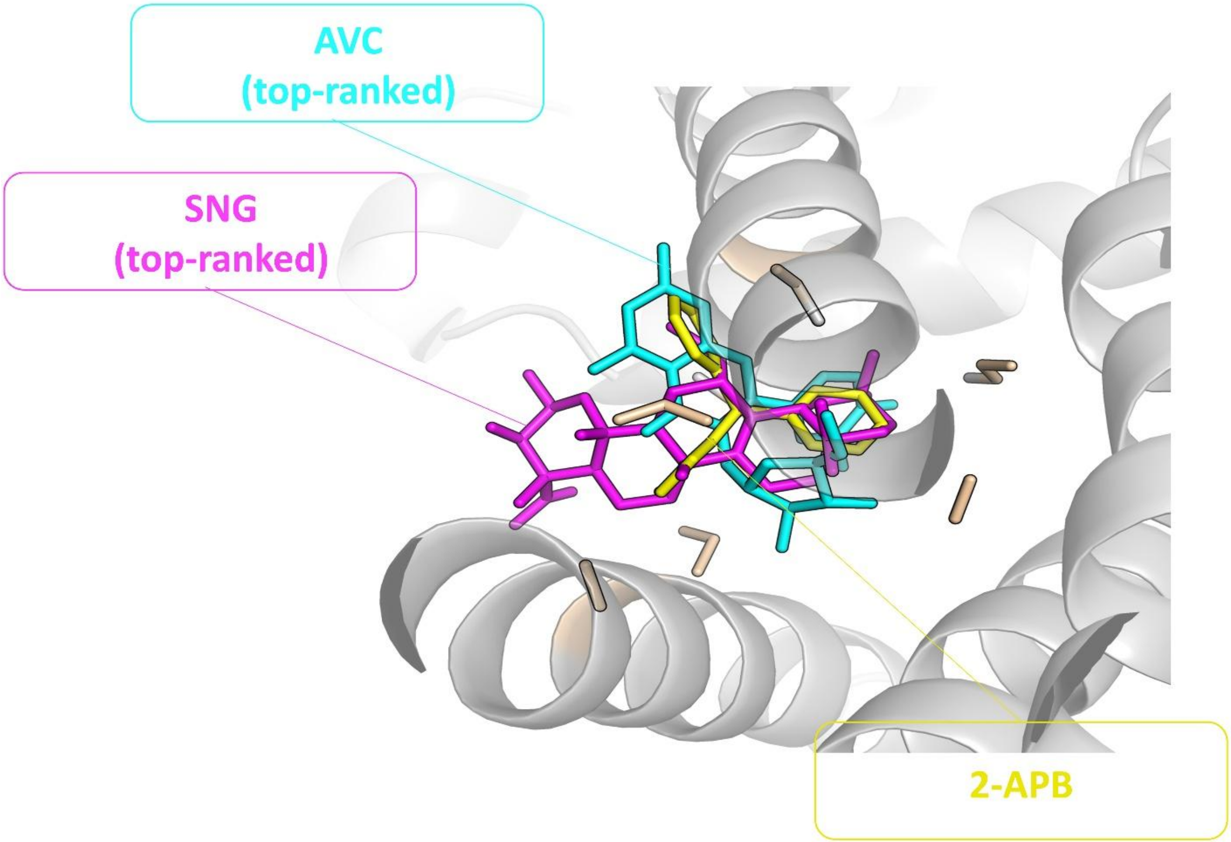

Supplementary Figure S3. Concentration–response effects of AVC and SNG on carvacrol-induced Ca^{2+}-dependent fluorescence responses in TRPV3-expressing HEK293 cells.

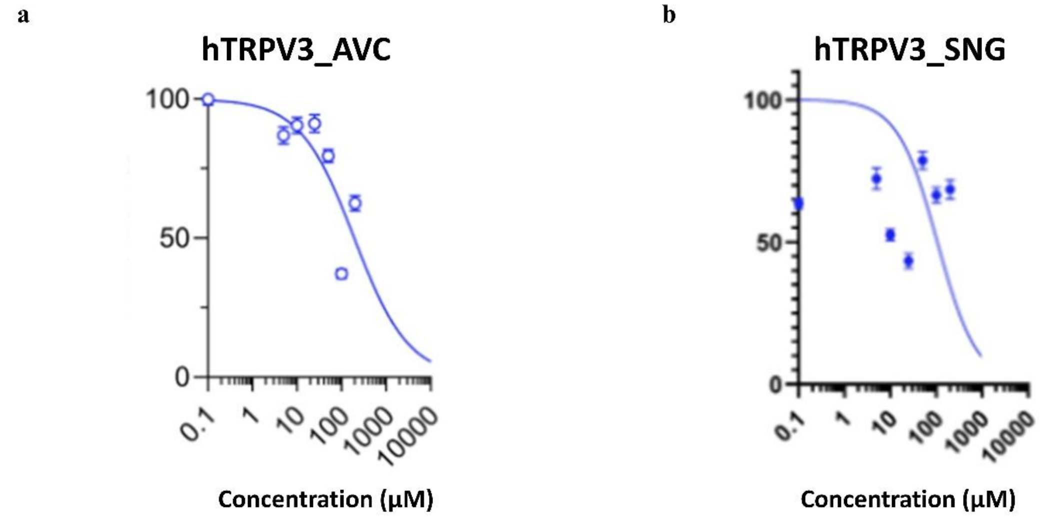

(A, B) Concentration–response analysis of AVC (A) and SNG (B) against carvacrol-induced Ca^{2+}-dependent fluorescence responses. AVC and SNG were tested at 0, 10, 25, 50, 100, and 200 μM. Data are presented as mean ± SD from three independent experiments (n = 3). Concentration–response curves were analyzed by nonlinear regression. The approximate apparent IC_{50} estimates were 192.9 μM (95% CI, 186.5–199.7 μM) for AVC and 105.7 μM (95% CI, 99.0–112.9 μM) for SNG. Because the tested concentration range did not adequately define both plateaus of the concentration–response curves, these values are interpreted as approximate apparent IC_{50} estimates rather than precise pharmacological IC_{50} values.

Supplementary Figure S4. Validation of siRNA-mediated TRPV3 knockdown in HaCaT cells.

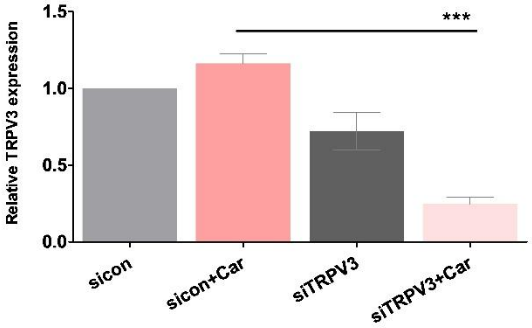

HaCaT cells were transfected with non-targeting control siRNA (siCon) or TRPV3-specific siRNA (siTRPV3), followed by carvacrol stimulation. TRPV3 mRNA expression was assessed by qRT-PCR. Under carvacrol-stimulated conditions, TRPV3 mRNA expression was reduced by approximately 79% in siTRPV3-transfected cells compared with siCon-transfected cells. Data are presented as mean ± SEM from three independent experiments. $^{***}p < 0.001$.
